# Epigenetic Clues to Better Understanding of the Asexual Embryogenesis *in planta* and *in vitro*

**DOI:** 10.3389/fpls.2019.00778

**Published:** 2019-06-18

**Authors:** Taras Pasternak, Denes Dudits

**Affiliations:** ^1^Institute of Biology II/Molecular Plant Physiology, Albert-Ludwigs-Universität Freiburg, Freiburg, Germany; ^2^Biological Research Centre, Institute of Plant Biology, Hungarian Academy of Sciences, Szeged, Hungary

**Keywords:** cellular differentiation, callus, totipotency, reprogramming, transcripts, division, stress

## Highlights

- One of most intriguing questions in developmental plant biology, is how the cellular totipotency is generated, and results in the asexual embryogenesis.
- Hormonal and stress signals play a key role in initiation of the embryogenic pathway by activation of cell division in somatic cells *in planta* and in *in vitro* cultured cells
- DNA hypomethylation or histone acetylation as epigenetic events activate expression of specific transcription factor, hormonal or developmental genes being responsible for totipotent stage.
- Ectopic expression of specific developmental genes can trigger somatic embryogenesis in vegetative plant organs.
- Level of DNA methylation in dedifferentiated callus tissues is lowered during embryogenesis.
- Epigenetic reprogramming is reflected by significant changes in transcript profiles during callus induction and somatic embryogenesis.

## Differentiation of Embryos or Plantlets From Vegetative Organs *in Planta*

**Figure 1:**
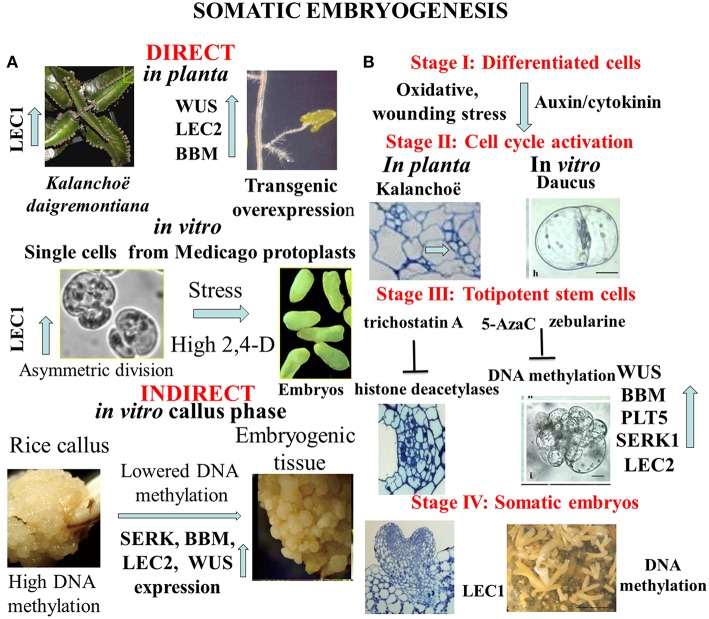
**(A)** Complexity of alternative pathways in the development of somatic embryos. *In planta* direct somatic embryogenesis can result in small plantlets appearing on margin of *Kalanchoe* leaves, or in root tips overexpression of transcription factor genes as WUSCHEL (WUS); LEAFY COTYLEDON 2 (LEC2), BABY BOOM (BBM) can trigger embryo formation. *In vitro* asymmetric cell division in protoplast-derived cells exposed to high dose of synthetic auxin (2,4-D-dichlorophenoxy acetic acid) or stress signals initiates the embryogenic pathway. Frequently somatic embryogenesis occurs in callus tissues representing undifferentiated pluripotent stem cells with hypermethylation of DNA that is lowered in pro-embryogenic cells. **(B)** Representative stages of somatic embryogenesis in Kalanchoë or Daucus somatic cells in relation to hypomethylation of DNA or acetylation of histone proteins. Reactivation of cell division cycle is a prerequisite for cellular reprogramming. Trichostatin A as inhibitor of histone deacetylases or 5-azacytidine/ zebularine as inhibitors of DNA methylation can generate chromatin structure to activate expression of specific developmental genes that are involved in formation of totipotent somatic plant cells. The end products are somatic embryos to be used in micropropagation or in molecular breeding (Dudits et al., 1991; Zuo et al., 2002; Garcês et al., 2007; Wani et al., 2011; Grzebelus et al., 2012; Guo et al., 2015; Zhu, 2017).

Formation of numerous buds and small plantlets on leaf margin of *Kalanchoe daigremontiana* is a peculiar developmental event in the plant kingdom (Figure 1A, Garcês et al., 2007). These organogenic or embryogenic processes start with cell divisions as responses to wounding or hormonal signals (Stage I) shown by Figure 1B (Guo et al., 2015; Zhu, 2017). Under formation of meristematic regions in *Kalanchoe* leaves, the chromatin status activates expression of specific key regulator and marker genes of both organogenesis (SHOOT MERISTEMLESS, STM) and embryogenesis (LEAFY COTYLEDON1, LEC1, and EMBRYONIC TEMPORAL REGULATOR, FUSCA3, Garcês et al., 2007). Suppression subtractive hybridization studies revealed that the overexpression of a large number (390) of unigenes in the asexual reproduction of *K*. *daigremontiana* (Zhong et al., 2013). Figure 1B highlights common cellular and molecular events in different stages of transition from somatic to embryogenic cell fate. In both cases (in Daucus: Grzebelus et al., 2012; Kalanchoe: Guo et al., 2015) hormonal and stress factors are involved in induction of cell division and cellular re-programming. However, the physiological machinery as well as epigenetic changes linked with these processes have been preferentially investigated in embryogenesis initiated from somatic cells. More recently, another plant species *Rhynia gwynne*-*vaughanii* was found to be capable for plant regeneration *in planta* (Kearney et al., 2016).

## *In vitro* Resetting Epigenetic Memory of Embryogenic Pathway Without or With Callus Induction

Thanks to the very intensive research from middle of last century, the somatic embryogenic pathway was observed in very different *in vitro* culture systems (Figure 1A). We see as a breakthrough in developmental biology when somatic embryo formation from root tips was observed in one of the activation tagged Arabidopsis mutants. It turned out that ectopic expression of WUSCHEL (WUS), a homeodomain protein in transgenic Arabidopsis plants caused embryo development from this vegetative organ (Figure 1A, Zuo et al., 2002). Similarly, overexpression of LEAFY COTYLEDON2 (LEC2) gene is sufficient to trigger the formation of somatic embryos from vegetative tissues (Stone et al., 2001). High number of somatic embryos was formed on the scutella of transgenic maize plants overexpressing transcription factors BABY BOOM (BBM) and WUSCHEL2 (WUS2) under the control of specific promoters (Lowe et al., 2018).

In contrast to the direct embryo formation from somatic tissues, frequently the callus stage is a prerequisite for cellular reprogramming that insures shutting down “old” cell fates and permitting upregulation of “new” cell fates through changing chromatin stage [see review by Fehér et al. (2003) and Fehér (2019)]. Several investigations demonstrated that the cell re-programming is accompanied by significant changes in chromatin status (DNA methylation and histone methylation/acetylation) (for review see Birnbaum and Roudier, 2017; Lee and Seo, 2018). Majority of studies was devoted for re-programming of callus cells to initiate shoot formation. Mutations in key epigenetic genes encoding for DNA METHYLTRANSFERASE (MET1), KRYPTONITE (KYP) for the histone 3 lysine 9 (H3K9) METHYLTRANSFERASE, JMJ14 for the histone 3 lysine 4 (H3K4) DEMETHYLASE, and HISTONE ACETYLTRANSFERASE (HAC1) resulted in altered WUS expression and developmental rates of regenerated shoots *in vitro* (Li et al., 2011). Clear sign for modification of the epigenetic landscape is the hypermethylation at certain genes in rice callus that was detected in CHH sequence contexts, at the promoter region of genes (Figure 1A, Stroud et al., 2013). Since transcriptional repression is associated with hypermethylation of DNA as a first step in developmental reprogramming, the callus stage can erase gene expression pattern by higher number of down-regulated genes (373) than the up-regulated ones (241) during callus formation from Arabidopsis root explants (Che et al., 2006). Callus formation is dependent on histone deacetylation shown by treatment of Arabidopsis leaf explants with trichostatin A (Lee et al., 2016). In addition, demethylation of H3K27me3 is critical for acquisition of callus formation from Arabidopsis leaves (He et al., 2012; Lee et al., 2018). Deposition of histone variant, H2A.Z strongly correlates with the gene activation mark H3K4me3 and genes regulated by H2A.Z may be related to environmental responses, chromatin assembly and cell cycle in callus representing undifferentiated pluripotent stem cells (Zhang et al., 2017).

However, these results cannot be directly extrapolated to reprogramming of the differentiated somatic cells to become embryogenic, there is strong experimental support for involvement of chromatin structure in this unique developmental event. Karim et al. (2018) reported that somatic embryogenesis in *Boesenbergia rotunda* (L.) was linked with relatively higher expression of *SOMATIC EMBRYOGENESIS RECEPTOR*-*LIKE KINASE* (SERK), BBM, LEC2, and WUS genes and lower level of DNA methylation. The 5-Azacytidine (5-AzaC), an inhibitor of DNA methylation was shown to stimulate somatic embryogenesis in *Pinus pinaster, Brassica napus, Hordeum vulgare*, and *Theobroma cacao* cultures (see review by Osorio-Montalvo et al., 2018). In non-embryogenic cotton calluses, inhibition of the DNA methylation by using zebularine treatment increased the number of embryos (Li et al., 2018). Stress responsive genes as heat shock gene can be activated during embryogenic induction in cultured alfalfa callus cells (Györgyey et al., 1991).

## Re-Programming of Terminally Differentiated Cells-Derived From Leaf Protoplasts

So far the majority of the investigations on cell re-programming was performed at level of multicellular structure. In order to avoid complexity of plant tissue in which even neighboring cells have different physiological/molecular status, homogenous population of leaf protoplast-derived cells can serve as an optimal experimental material for studies on cell re-programming. Selected Medicago genotypes offer an optimal experimental system for detailed analysis of cellular reprogramming, especially in protoplasts cultures. Comparison of embryogenic and non-embryogenic cells can provide deeper insight both at cellular and molecular levels. One experimental system was based on alfalfa (*Medicago sativa* L.) leaf protoplasts (A2 line) with unique capability to generate totipotent cells from isolated mesophyll protoplasts in culture medium with high dose (≤1 mg/L) of exogenous auxin analogy, 2,4-D-dichlorophenoxy acetic acid (2,4-D) (Dudits et al., 1991; Figure 1A, Bögre et al., 1990). During protoplasts re-programming size of DAPI stained nuclei was significantly increased, especially on the medium with high 2,4-D concentration, that can reflect more relaxed chromatin (Pasternak et al., 2000). The embryogenic alfalfa cells could be characterized by earlier cell division, a more alkalic vacuolar pH, and non-functional chloroplasts (Pasternak et al., 2002). In parallel, in the embryogenic cells 38 up-regulated transcripts preferentially from stress responsive genes could be identified by PCR-based cDNA subtraction approach (Domoki et al., 2006). The LEC1, embryogenic gene exhibited more than seven-fold higher expression in the presence of the high 2,4-D concentration relative to cells grown in medium with low 2,4-D. This activation of LEC1 gene in embryogenic cells is linked to the reactivation of cell cycle and generation of polarity by asymmetric cell division (Figure 1A). These events can be clearly monitored in protoplasts cultures where auxin and oxidative stress factors can activate cyclin-dependent kinase complexes and induction of S-phase (Ötvös et al., 2005; Pasternak et al., 2007, 2014; Fehér et al., 2008; Fehér, 2015). Non-embryogenic cell types contain big lytic vacuole (acidic one) but embryogenic cells have numerous storage protein (more alkaline). These characteristics can be seen in the de-differentiated stem cells *in planta* (Pasternak et al., 2002). The embryogenic genotype of alfalfa exhibited highly dense cytoplasm, with reduced cell expansion, and frequent asymmetric cell division (Bögre et al., 1990; Dudits et al., 1991). Dijak and Simmonds (1988) reported that in embryogenic alfalfa cells microtubule strands developed more rapidly, and microtubules were finer and more branched than in non-embrygenic protoplasts. Important signs for the embryogenic re-programming of somatic cells can be recognized during the first cell division. The gap between initiation of culture and first DNA replication events—what is much longer as normal G1 phase- and detection of increased nuclei size and stainability suggests significant role for chromatin relaxation in the process of cell cycle activation. This step is a key event in re-programming cells to reactivate division requiring auxin in the culture media (Pasternak et al., 2000, 2002).

## Conclusions

Plasticity of cellular differentiation in plants is not only a very exciting biological phenomenon, but it is an important component in tissue culture-based propagation systems or in transgenic and genome editing technologies. In the present opinion paper we demonstrate that defined *in vitro* conditions with hormonal or stress effects can generate chromatin status that insures activation of specific transcription factor (WUS; LEC1, 2; BBM) genes of embryogenic program. Alternatively, transgenic overexpression of these genes can also initiate similar developmental pathway in variety of cell types. Recent publications using inhibitor of histone deacetylation or DNA methylation provide strong support for the direct involvement of chromatin status in cellular reprogramming including callus formation and asexual embryogenesis (Lee et al., 2016; Li et al., 2018; Osorio-Montalvo et al., 2018). The key role of cell division in somatic embryogenesis could be clearly shown by using of protoplast-derived homogenous cell populations for molecular and structural studies. The present analysis mainly based on some “model” plants exhibiting somatic embryogenesis as specific trait. The present progress in discovering the underlying molecular and cellular events (see review by Fehér, 2019) is expected to extent this phenomenon to other plant species also with agronomic significance.

## Author Contributions

All authors listed have made a substantial, direct and intellectual contribution to the work, and approved it for publication.

## Contribution to the Field Statement

Generation of totipotent stage in differentiated plant cells through molecular and cellular reprogramming attracts significant interest in the field of plant science. Plasticity of cellular differentiation/de-differentiation is an important component in the tissue culture-based propagation systems or in transgenic and genome editing technologies. Here we point out common features in plant cell re-programming from different explants with focus on the role of epigenetic mechanism and activities of developmental genes. We outline advantages of the use of protoplast-derived homogenous cell populations for the auxin/stress concentration-dependent induction of embryogenic program.

### Conflict of Interest Statement

The authors declare that the research was conducted in the absence of any commercial or financial relationships that could be construed as a potential conflict of interest.
